# lncRNA *TUG1* mediates palmitic acid-induced neuronal lipotoxic injury via the miR-449a-5p/caspase-3 and Akt/GSK-3β axes

**DOI:** 10.3892/ijmm.2026.5966

**Published:** 2026-08-25

**Authors:** Ya-Dong Wei, Peng-Quan Chen, Xin Zheng, Le-Qi Wu, Xin-Yi Wang, Xin-Ran Gao, Jin-Fang Ge

**Affiliations:** 1 Department of Basic and Clinical Pharmacology, School of Pharmacy, Anhui Medical University, Hefei, Anhui 230032, P.R. China; 2 Anhui Provincial Laboratory of Inflammatory and Immunity Disease, Anhui Institute of Innovative Drugs, Hefei, Anhui 230032, P.R. China; 3 The Key Laboratory of Anti-inflammatory and Immune Medicine, Ministry of Education, Anhui Medical University, Hefei, Anhui 230032, P.R. China

**Keywords:** lncRNA *TUG1*, miR-449a-5p, Caspase-3, palmitic acid, Akt, GSK-3β pathway

## Abstract

A close relationship exists between excessive lipids and structural and functional brain dysfunction, with the long non-coding (lncRNA)-microRNA (miR)-mRNA network having an emerging role. Given the increasingly recognized role of the lncRNA taurine upregulated gene 1 (*TUG1*) in metabolic and neurodegenerative diseases, the present study investigated its function and mechanism in palmitic acid (PA)-induced neuronal injury. Results showed that lncRNA *TUG1* expression was elevated in PA-treated HT-22 and SH-SY5Y cells. Moreover, cell-based *in vitro* detection including reverse transcription-quantitative polymerase chain reaction, western blotting and lactate dehydrogenase cytotoxicity assays demonstrated a positive association between lncRNA *TUG1* expression and lactate dehydrogenase release and cleaved caspase-3 levels, whereas a negative association was observed with the expression levels of Bcl-2 and synaptic proteins (synapsin-1, synaptotagmin-1 and brain-derived neurotrophic factor) and the ratios of phosphorylated (p-) Akt/Akt and p-GSK-3β/GSK-3β. The downregulation of lncRNA *TUG1* reversed PA-induced damage in HT-22 cells. Bioinformatics and dual-luciferase assays identified miR-449a-5p as the direct target of lncRNA *TUG1*. Rescue experiments revealed that miR-449a-5p mediated the effects of lncRNA *TUG1* by targeting caspase-3 via the Akt/GSK-3β pathway. Collectively, these findings establish a causal axis in which lncRNA *TUG1* derepresses caspase-3 and suppresses Akt/GSK-3β signaling by sponging miR-449a-5p, thereby driving neuronal lipotoxic injury.

## Introduction

Hyperlipidemia is a common metabolic disorder characterized by elevated serum cholesterol and triglycerides. Beyond its well-established role in cardiovascular disease, increasing evidence has linked hyperlipidemia to cognitive impairment and all-cause dementia (1,2). Patients with hyperlipidemia have a notably higher risk of developing Alzheimer's disease (AD) (3,4). Furthermore, elevated levels of free fatty acids, particularly palmitic acid (PA), have been implicated as key pathogenic mediators (2,5). PA can cross the blood-brain barrier and accumulate in brain tissues, where it triggers lipotoxic injury including oxidative stress, apoptosis and synaptic dysfunction (6). However, the molecular mechanisms through which PA induces neuronal damage remain unclear.

The hippocampus, a brain region that is essential for learning and memory, is particularly vulnerable to metabolic stress. Elevated free fatty acid levels are correlated with reduced hippocampal volume and impaired episodic memory scores (7,8). Our previous studies have consistently demonstrated that excessive lipids directly damage hippocampal neurons. In animal models of non-alcoholic fatty liver disease (NAFLD) or type 2 diabetes mellitus (T2DM), hyperlipidemia, hippocampal neuronal apoptosis, synaptic protein loss and cognitive deficits were observed (9-11). *In vitro*, it was found that PA treatment of HT-22 cells induced lipid accumulation, insulin resistance and the downregulation of synaptic plasticity related proteins such as synapsin-1 and brain-derived neurotrophic factor (BDNF) (12). These results indicate that PA exposure leads to substantial injury to hippocampal neurons. However, the upstream molecular mechanisms remain largely unknown.

Long non-coding RNAs (lncRNAs) have emerged as critical regulators of gene expression under both physiological and pathological conditions. Through mechanisms such as acting as competing endogenous RNAs (ceRNAs), lncRNAs can sponge microRNAs (miRNAs/miRs) and modulate the expression of downstream target genes (13). Dysregulated lncRNA expression is increasingly recognized in the context of metabolic disorders and neurodegeneration. For instance, lncRNA *MEG3* attenuates hyperglycemia-induced damage by enhancing the mitochondrial translocation of HSP90A in primary hippocampal neurons (14), while lncRNA *MALAT1* is involved in oxidative stress responses in diabetic encephalopathy (15). However, the specific lncRNAs that govern PA-induced neuronal lipotoxicity have not been systematically explored.

The taurine upregulated lncRNA *TUG1* is the first non-coding RNA identified in mouse retinal cells. In addition to its critical role in retinal development and the maintenance of normal photoreceptors (16), lncRNA *TUG1* serves as a potential mediator of metabolic-neural crosstalk (17). Elevated lncRNA *TUG1* expression has been detected in the serum or cerebrospinal fluid from patients with temporal lobe epilepsy (18), Parkinson's disease (19) and AD (20), and is closely associated with cellular apoptosis and inflammatory responses; it sponges miR-145a-5p and aggravates myocardial fibrosis in a diabetic cardiomyopathy mouse model by upregulating *Cfl2* (21). Additionally, the knockdown of lncRNA *TUG1* can inhibit the apoptosis of hippocampal neurons in AD by upregulating miR-15a and downregulating *ROCK1* expression (22). Similarly, beneficial effects have also been found for lncRNA *TUG1* downregulation in BV_2_ cells against the challenge of lipopolysaccharides/interferon-γ via an involvement in inhibiting glycolysis and facilitating the shift of microglial glucose metabolism from glycolysis to oxidative phosphorylation (23). Previous evidence indicates that lncRNA *TUG1* also contributes to hepatic steatosis and pancreatic β cell dysfunction (24,25), thus suggesting a broader role in lipotoxicity. Nevertheless, whether lncRNA *TUG1* participates in PA-induced neuronal lipotoxic injury in the central nervous system has not yet been investigated.

The present study aimed to investigate whether PA-induced neuronal injury involves lncRNA *TUG1* dysregulation and to explore the underlying molecular mechanisms. Specifically, the present study examined the effects of PA on lncRNA *TUG1* expression in HT-22 and SH-SY5Y cells and assessed the functional consequences of lncRNA *TUG1* knockdown on PA-induced apoptosis, synaptic dysfunction and lipid peroxidation. To elucidate the downstream mechanism, it was further explored whether miR-449a-5p mediates the effects of lncRNA *TUG1* by targeting caspase-3 and modulating the Akt/GSK-3β pathway. This was addressed by gain- and loss-of-function approaches for miR-449a-5p, combined with pharmacological inhibition of Akt. Through these approaches, the present study sought to characterize the lncRNA *TUG1*/miR-449a-5p/caspase-3 and Akt/GSK-3β axes in PA-induced neuronal lipotoxic injury.

## Materials and methods

### Cell culture

The murine hippocampal neuronal cell line HT-22 (cat. no. SNL-202; Wuhan Shangen Biotechnology Co., Ltd.) and the human neuroblastoma cell line SH-SY5Y (cat. no. SNL-092; Wuhan Shangen Biotechnology Co., Ltd.) were cultured in high-glucose Dulbecco's Modified Eagle Medium (DMEM; cat. no. SH30022; HyClone; Cytiva) supplemented with 10% fetal bovine serum (FBS; cat. no. FCS500; Suzhou ExCell Biotechnology Co., Ltd.) at 37°C in a humidified incubator with 5% CO_2_. The SH-SY5Y cell line was authenticated by Wuhan Shangen Biotechnology Co., Ltd. using short tandem repeat (STR) profiling with the PowerPlex^®^ 18D system. The STR profile showed a 100% match with the SH-SY5Y reference profile in the ExPASy Cellosaurus database (CVCL_0019), and no interspecies cross-contamination was detected. The 293T cells used in the present study were purchased from Wuhan Servicebio Technology Co., Ltd. (cat. no. STCC10301) and were maintained in the same culture medium and conditions as aforementioned. The cells were passaged three to five times per week. Exponentially growing cells were seeded into a 6-well plate at a density of 5×10^5^ cells/well and allowed to reach 60% confluency. According to a previous study, PA (100 *μ*M) was used to stimulate HT-22 or SH-SY5Y cells to establish a hyperlipidemic injury model (26).

### 3-(4,5-Dimethylthiazol-2-yl)-2,5-diphenyltetrazolium bromide (MTT) and lactate dehydrogenase (LDH) assays

The MTT assay was performed according to the manufacturer's instructions (cat. no. M8180; Beijing Solarbio Science & Technology Co., Ltd.). Briefly, after treatment with various concentrations of PA (25, 50, 100, 200, 400 and 800 *μ*M) for 24 h at 37°C, cells were washed twice with 1X phosphate-buffered saline (PBS) solution and then incubated in complete medium supplemented with 0.5 mg/ml MTT for 4 h. After incubation, the supernatant was removed from each well and 100 *μ*l of Formazan Dissolution Solution (provided in the MTT kit) was added. The cells were gently mixed and incubated in a constant temperature incubator at 37°C until all purple crystals had dissolved. The absorbance was measured at 570 nm using a microplate reader.

Cytotoxicity was evaluated by quantifying the release of LDH from cell supernatants using the Lactate Dehydrogenase Cytotoxicity Assay Kit (cat. no. C0016; Beyotime Biotechnology) according to the manufacturer's instructions.

### Cell transfection

For transfection, HT-22 cells were seeded into 6-well plates at a density of 5×10^5^ cells/well and randomly divided into different groups (n=3 for each group). When the cells grew to 40-60% confluency, transfection was performed using Lipofectamine™ 2000 Transfection Reagent (cat. no. 11668027; Thermo Fisher Scientific, Inc.) according to the manufacturer's instructions. Briefly, the transfection reagent and nucleotides [small interfering RNA targeting lncRNA *TUG1* (si-lncRNA *TUG1*), miR-449a-5p mimics, miR-449a-5p inhibitor or the corresponding negative controls (NCs)] were each diluted in 200 *μ*l serum-free DMEM, mixed gently and incubated for 10 min at room temperature. The mixtures were then added to the cells, followed by incubation at 37°C for 6 h. At 6 h post-transfection, the medium was replaced with complete DMEM containing 100 *μ*M PA and the cells were cultured at 37°C for a further 24 h prior to subsequent analyses. Cells were subsequently collected for RNA extraction, protein extraction, apoptosis detection or lipid peroxidation (LPO) assays. All nucleotides were synthesized by Shanghai GenePharma Co., Ltd. All transfections were performed with 50 nM nucleotide.

The nucleotide sequences were as follows: siRNA-1, 5'-GCCAGGUUUAUUCCAUAAATT-3'; siRNA-2, 5'-GGACUUGCAACCUGGUUAUTT-3'; siRNA-3, 5'-GCCUGUUCUUCUAGCUUAATT-3'; siNC sense, 5'-UUCUCCGAACGUGUCACGUTT-3' and antisense, 5'-ACGUGACACGUUCGGAGAATT-3'; mimics sense, 5'-UGGCAGUGUAUUGUUAGCUGGU-3' and anti-sense, 5'-CAGCUAACAAUACACUGCCAUU-3'; inhibitor, 5'-ACCAGCUAACAAUACACUGCCA-3'; miR inhibitor NC, 5'-CAGUACUUUUGUGUAGUACAA-3'; miR mimic NC sense, 5'-UUCUCCGAACGUGUCACGUTT-3' and antisense, 5'-ACGUGACACGUUCGGAGAATT-3'.

### Measurement of malondialdehyde (MDA), LPO and 4-hydroxynonenal (4-HNE)

To examine whether LPO levels were altered, the levels of MDA (cat. no. S0131M; Beyotime Biotechnology) and LPO (cat. no A106-1; Nanjing Jiancheng Bioengineering Institute) were examined separately. Briefly, cells were collected after performing the corresponding treatments. Cell samples were subjected to repeated rapid freeze-thawing five times in liquid nitrogen and 37°C and were assayed separately according to the manufacturer's requirements.

In addition, the concentration of 4-HNE in HT-22 cell homogenates was measured via Enzyme-Linked Immunosorbent Assay using a commercial kit (cat. no. JYM1109Mo; Wuhan GeneBeauty Biotechnology Co., Ltd.) according to the manufacturer's instructions.

### Cell apoptosis analysis

HT-22 cell apoptosis was detected using Annexin V-FITC/propidium iodide (PI) double staining (cat. no. BB-4101; Shanghai Bestbio Biotechnology Co., Ltd.). After removing the medium, the HT-22 cells in each treatment group were stained with Annexin V-FITC and PI staining solution in the dark at 4°C for 15 min. The cells were then washed twice with PBS. Apoptotic cells were analyzed using a CytoFLEX flow cytometer (Beckman Coulter, Inc.) with FlowJo v10.8.1 software (FlowJo LLC; BD Biosciences).

### Bioinformatics analysis

The sequence and basic characteristics of lncRNA *TUG1* were obtained from the National Center for Biotechnology Information (NCBI) database (https://www.ncbi.nlm.nih.gov/). The LncLocator 2.0 tool (http://www.csbio.sjtu.edu.cn/bioinf/lncLocator2/) was used to analyze the subcellular localization of the lncRNA *TUG1* (27). The binding sites between lncRNA/mRNA and miRNA were predicted using miRDB (http://mirdb.org/index.html) and RNA Interactome (http://www.rna-society.org/raid/) database online prediction software (28).

### Dual-luciferase reporter gene assay

Both wild-type (WT) and mutant (Mut) fragments of lncRNA *TUG1*, which span the predicted miR-449a-5p binding site, were designed and synthesized by General Biotechnology (Anhui) Co., Ltd., and subsequently cloned into the psiCHECK™-2 vector (Promega Corporation) downstream of the *Renilla* luciferase coding sequence. The Mut plasmid contained nucleotide substitutions within the core binding site to abrogate miRNA binding. Data S1 includes the sequences used for plasmid construction and verification.

293T cells were maintained in DMEM supplemented with 10% FBS. At 80-90% confluency, cells were co-transfected with either *TUG1*-WT or *TUG1*-Mut reporter plasmid, along with miR-449a-5p mimics or mimic NC, using Lipofectamine^®^ 2000 (Invitrogen; Thermo Fisher Scientific, Inc.). After 5 h, the transfection mixture was replaced with fresh complete medium. Cells were harvested 24 h post-transfection, and luciferase activities were measured using the Dual-Luciferase^®^ Reporter Assay System (Promega Corporation) on a BioTek Synergy 2 microplate reader. *Renilla* luciferase activity was normalized to firefly luciferase activity for each sample. All experiments were performed in triplicate.

### Reverse transcription-quantitative polymerase chain reaction (RT-qPCR)

Total RNA was extracted from samples using TRIzol reagent (cat. no. 15596018CN; Invitrogen; Thermo Fisher Scientific, Inc.) according to the manufacturer's instructions. Subsequently, 1 *μ*g of total RNA was reverse-transcribed into cDNA in a total reaction volume of 10 *μ*l using a reverse transcription kit (cat. no. AG11706; Accurate Biology) according to the manufacturer's instructions. The resulting cDNA was diluted 5-fold to a final volume of 50 *μ*l with nuclease-free water. qPCR was performed in a 10 *μ*l reaction system containing 3 *μ*l of diluted cDNA, 0.3 *μ*l of each forward and reverse primer (10 *μ*M) and 5 *μ*l of 2X SYBR Green SupTaq HS Premix (AG11762; Guangzhou Ruizhen Biotechnology Co., Ltd.). The thermocycling conditions were as follows: Initial denaturation at 95°C for 30 sec, followed by 40 cycles of denaturation at 95°C for 5 sec and annealing/extension at 60°C for 30 sec. All reactions were performed in triplicate on an ABI Prism 7000 sequence detection system (Applied Biosystems; Thermo Fisher Scientific, Inc.). β-actin was used as the internal reference for mRNA and lncRNA *TUG1*, whereas U6 was used for miR-449a-5p. Relative expression was calculated using the 2^−ΔΔCq^ method (29). The primer sequences for the mouse genes were as follows: lncRNA *TUG1* forward, 5'-CAAGAAACAGCAACACCAGAAG-3' and reverse, 5'-TAAGGTCCCCATTCAAGTCAGT-3'; caspase-3 forward, 5'-GAGCTTGGAACGGTACGCTA-3' and reverse 5'-CCGTACCAGAGCGAGATGA-3'; β-actin forward, 5'-AGTGTGACGTTGACATCCGT-3', and reverse, 5'-TGCTAGGAGCCAGAGCAGTA-3'; miR-449a-5p forward, 5'-AACACGTGTGGCAGTGTATTG-3'; miR-34a-5p forward, 5'-AACCGGTGGCAGTGTCTTAG-3'; miR-6378 forward, 5'-AACAAGTGGTTCACGGGAGGA-3'; miR-421 forward 5'-CGCATCAACAGACATTAATTGGGCGC-3'; miR-199a-3p forward 5'-CGCACAGTAGTCTGCACATTGGTTA-3'; U6 forward, 5'-GGAACGATACAGAGAAGATTAGC-3' and reverse 5'-TGGAACGCTTCACGAATTTGCG-3'. The universal reverse primer for all miR qPCR assays was 5'-GCGAGCACAGAATTAATACGAC-3'. The primer sequences for the human genes were as follows: lncRNA *TUG1* forward, 5'-TGAGCAAGCACTACCACCAG-3' and reverse, 5'-ACTCAGCAATCAGGAGGCAC-3'; β-actin forward 5'-GATGAGATTGGCATGGCTT-3' and reverse 5'-GTCACCTTCACCGTTCCAGT-3'.

### Western blotting

Western blotting was performed as described previously (30). Total protein from HT-22 and SH-SY5Y cells was extracted using RIPA lysis buffer (cat. no. P0013B; Beyotime Biotechnology) supplemented with a protease and phosphatase inhibitor cocktail (50X; cat. no. P1046; Beyotime Biotechnology) at a ratio of 50:1. After centrifugation at 13,500 × g for 30 min at 4°C, the supernatant was collected and the total protein concentration was quantified using the bicinchoninic acid protein concentration assay kit (cat. no. PC0020; Beyotime Biotechnology). The protein samples were mixed with 5X SDS-PAGE protein loading buffer (cat. no. P0015L; Beyotime Biotechnology) and heated in boiling water for 10 min. Cooled protein samples (30 *μ*g per lane) were separated on 10% SDS-PAGE gels and transferred onto polyvinylidene difluoride (PVDF) membranes. PVDF membranes were blocked with 5% skimmed milk in Tris-buffered saline with 0.05% Tween 20 for 2 h at room temperature, then incubated overnight at 4°C with the following antibodies: Anti-phosphorylated (p-)GSK-3β (ser9) (1:1,000; cat. no. 9323; Cell Signaling Technology, Inc.), anti-GSK-3β (1:1,000; cat. no. 9315; Cell Signaling Technology, Inc.), anti-p-Akt1 (Ser473) (1:1,000; cat. no. 44-623G; Thermo Fisher Scientific, Inc.), anti-Akt (1:1,000; cat. no. WL0003b; Wanleibio Co., Ltd.), anti-synaptotagmin-1 (1:1,000; cat. no. 4329; Cell Signaling Technology, Inc.), anti-BDNF (1:1,000; cat. no. ab108319; Abcam), anti-Synapsin-1 (1:1,000; cat. no. 5297; Cell Signaling Technology, Inc.), anti-Bcl-2 (1:500; cat. no. WL01556; Wanleibio Co., Ltd.), anti-caspase-3/cleaved-caspase-3 (1:500; cat. no. WL02117; Wanleibio Co., Ltd.), anti-caspase-3 (1:1,000; cat. no. GB12532-50; Wuhan Servicebio Technology Co., Ltd.) and anti-β-actin (1:1,000; cat. no. TA-09; ZSGB-Bio; Beijing Zhongshan Jinqiao Biotechnology Co., Ltd.). The PVDF membranes were then incubated with a horseradish peroxidase-conjugated goat anti-rabbit (1:5,000; cat. no. ab6721; Abcam), or goat anti-mouse secondary antibody (1:5,000; cat. no. ab6789; Abcam) at room temperature for 1 h. Protein signals were detected using SuperSignal West Pico PLUS chemiluminescent substrate (cat. no. 34577; Thermo Fisher Scientific, Inc.), and the images were processed and analyzed using ImageJ software (version 1.52a; National Institutes of Health).

### Akt inhibitor VIII treatment

Akt inhibitor VIII (cat. no. SF2784-10 mM; Beyotime Biotechnology) was dissolved in dimethyl sulfoxide (DMSO) to prepare a stock solution. The final concentration of DMSO in the culture medium was maintained below 0.1% to avoid solvent-induced cytotoxicity. For pharmacological inhibition of Akt, HT-22 cells were treated with 5 *μ*M Akt inhibitor VIII simultaneously with 100 *μ*M PA for 24 h at 37°C, with the administration protocol set with reference to a previous study (31). The experimental groups included control (untreated), PA only, PA plus NC, PA plus si-TUG1, PA plus Akt inhibitor VIII alone, PA plus si-TUG1 combined with Akt inhibitor VIII, and PA plus si-TUG1 combined with DMSO vehicle control. The same grouping strategy was also applied to miR-449a-5p mimics in place of si-TUG1. After the treatment, cells were harvested for western blot analysis of p-Akt, total Akt, p-GSK-3β, total GSK-3β, cleaved caspase-3, total caspase-3, Bcl-2, synapsin-1, synaptotagmin-1 and BDNF.

### Statistical analysis

Data are expressed as mean ± standard error of the mean. Data were analyzed using SPSS 23.0 (IBM Corp.) and GraphPad Prism 8.0 software (Dotmatics). All data were first tested for normality using the Shapiro-Wilk test. Unpaired Student's t-test was used for comparisons between two groups. One-way analysis of variance (ANOVA) was performed to compare three or more groups. When the result of the overall ANOVA was statistically significant, the Bonferroni post hoc test was used for pairwise comparisons. Correlation analyses were performed using Pearson's correlation test. P<0.05 was considered to indicate a statistically significant difference.

## Results

### lncRNA TUG1 is upregulated in PA-induced neuronal injury and may be correlated with apoptosis and Akt/GSK-3β pathway dysfunction in HT-22 cells

**Figure 1:**
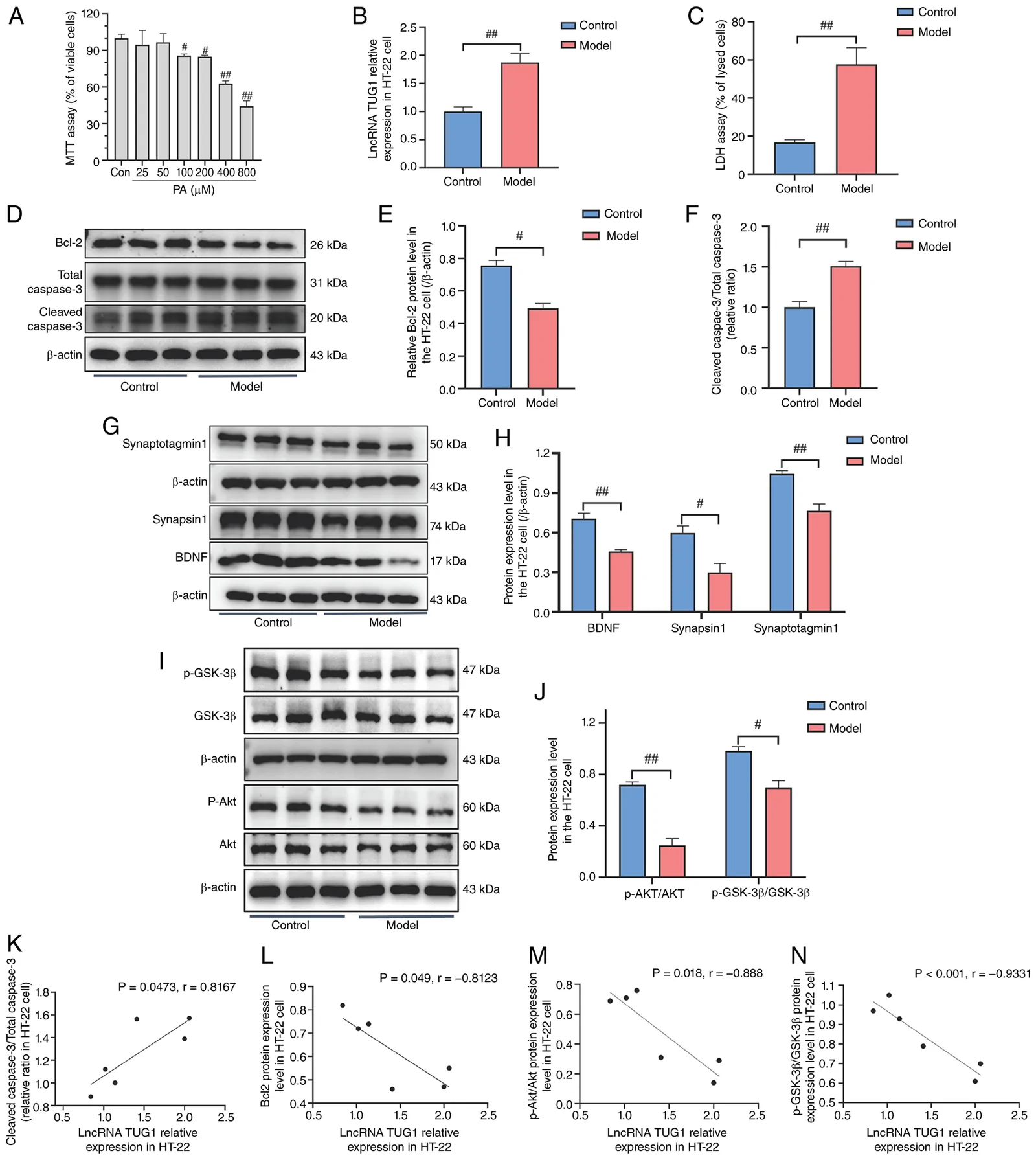
Effects of PA challenge on lncRNA *TUG1* expression as well as apoptosis, synaptic plasticity and Akt/GSK-3β pathway in HT-22 cells. (A) MTT results of HT-22 cells challenged with different concentrations of PA. (B) Expression of lncRNA *TUG1* in HT-22 cells. (C) LDH rates in HT-22 cells. (D) Representative western blotting bands of Bcl-2, cleaved caspase-3 and total caspase-3. Semi-quantification of (E) Bcl-2 protein levels and (F) the cleaved caspase-3 to total caspase-3 ratio. (G) Representative western blotting bands of Synapsin-1, Synaptotagmin-1 and BDNF. (H) Semi-quantification of the Synapsin-1, Synaptotagmin-1 and BDNF protein levels. (I) Representative western blotting bands of Akt, p-Akt, GSK-3β and p-GSK-3β. (J) Semi-Quantification of the p-Akt/Akt and p-GSK-3β/GSK-3β ratios. Correlation between lncRNA TUG1 expression and (K) the cleaved caspase-3/total caspase-3 ratio, (L) Bcl-2 protein levels, (M) the p-Akt/Akt ratio and (N) the p-GSK-3β/GSK-3β ratio. All treatments were performed using 100 *μ*M PA for 24 h. Representative western blotting bands are from three independent biological replicates per group for both the control and PA-treated conditions. The data are presented as the mean ± SEM, with n=3 for each group. ^#^P<0.05, ^##^P<0.01 compared with the control group. BDNF, brain-derived neurotrophic factor; LDH, lactate dehydrogenase; lncRNA, long non-coding RNA; MTT, 3-(4,5-dimethylthiazol-2-yl)-2,5-diphenyltetrazolium bromide assay; PA, palmitic acid; p, phosphorylated; TUG1, taurine upregulated gene 1.

To explore the role of lncRNA *TUG1* in PA-induced neuronal injury, HT-22 cells were treated with a concentration gradient of PA (25-800 *μ*M) for 24 h. MTT assay results showed that treatment with 100 *μ*M PA for 24 h significantly reduced HT-22 cell viability compared with the control group, whereas higher concentrations (200-800 *μ*M) caused progressively greater cytotoxicity (Fig. 1A). Therefore, 100 *μ*M PA was selected as the optimal concentration for establishing the neuronal injury model in subsequent experiments, as it induced significant but submaximal injury, consistent with a previous study (26). Thus, 100 *μ*M PA treatment for 24 h was selected as the experimental condition for subsequent studies in HT-22 cells. In this model, PA exposure significantly upregulated lncRNA *TUG1* expression and increased LDH release, indicating neuronal damage (Fig. 1B and C). Apoptosis-related protein assays showed that PA treatment significantly increased the cleaved caspase-3 to total caspase-3 ratio and reduced the expression of anti-apoptotic protein Bcl-2 (Fig. 1D-F), indicating that PA activated the apoptotic pathway in HT-22 cells. Given that Akt/GSK-3β signaling is a core pro-survival pathway in neurons and has been implicated in lipotoxic neuronal injury (32), it was next examined whether PA exposure disrupts this signaling axis and its downstream synaptic targets. PA stimulation significantly downregulated the protein levels of synapsin-1, synaptotagmin-1 and BDNF (Fig. 1G and H) and those of the Akt/GSK-3β signaling pathway, as reflected by the decreased ratios of p-Akt/Akt and p-GSK-3β/GSK-3β (Fig. 1I and J). These data indicated that PA impaired synaptic function and inhibited Akt/GSK-3β signaling.

Given that the expression levels of molecules causally involved in disease pathogenesis often correlate quantitatively with disease severity and key pathological indicators (33), exploratory Pearson correlation analysis was performed to systematically investigate the associations between lncRNA *TUG1* expression levels and PA-induced molecular alterations. The data used for these analyses were obtained from PA-treated HT-22 cells. LncRNA *TUG1* expression was measured as shown in Fig. 1B, and the cleaved caspase-3/total caspase-3 ratio (Fig. 1F), Bcl-2 protein levels (Fig. 1E) and p-Akt/Akt and p-GSK-3β/GSK-3β ratios (Fig. 1J) were determined accordingly. Pearson correlation analysis revealed that lncRNA *TUG1* expression was positively correlated with the cleaved caspase-3/total caspase-3 ratio and negatively correlated with the Bcl-2, p-Akt/Akt and p-GSK-3β/GSK-3β ratios (Fig. 1K-N), suggesting that lncRNA *TUG1* may participate in PA-induced neuronal injury by regulating apoptosis and synaptic function. Similar results were observed in SH-SY5Y cells (Fig. S1A-L), further supporting the universal role of lncRNA *TUG1* in lipid toxicity-induced neuronal damage.

### Knocking down lncRNA TUG1 in HT-22 cells alleviates PA-induced dysfunctional changes in apoptosis, synaptic plasticity, Akt/GSK-3β pathway and LPO

**Figure 2:**
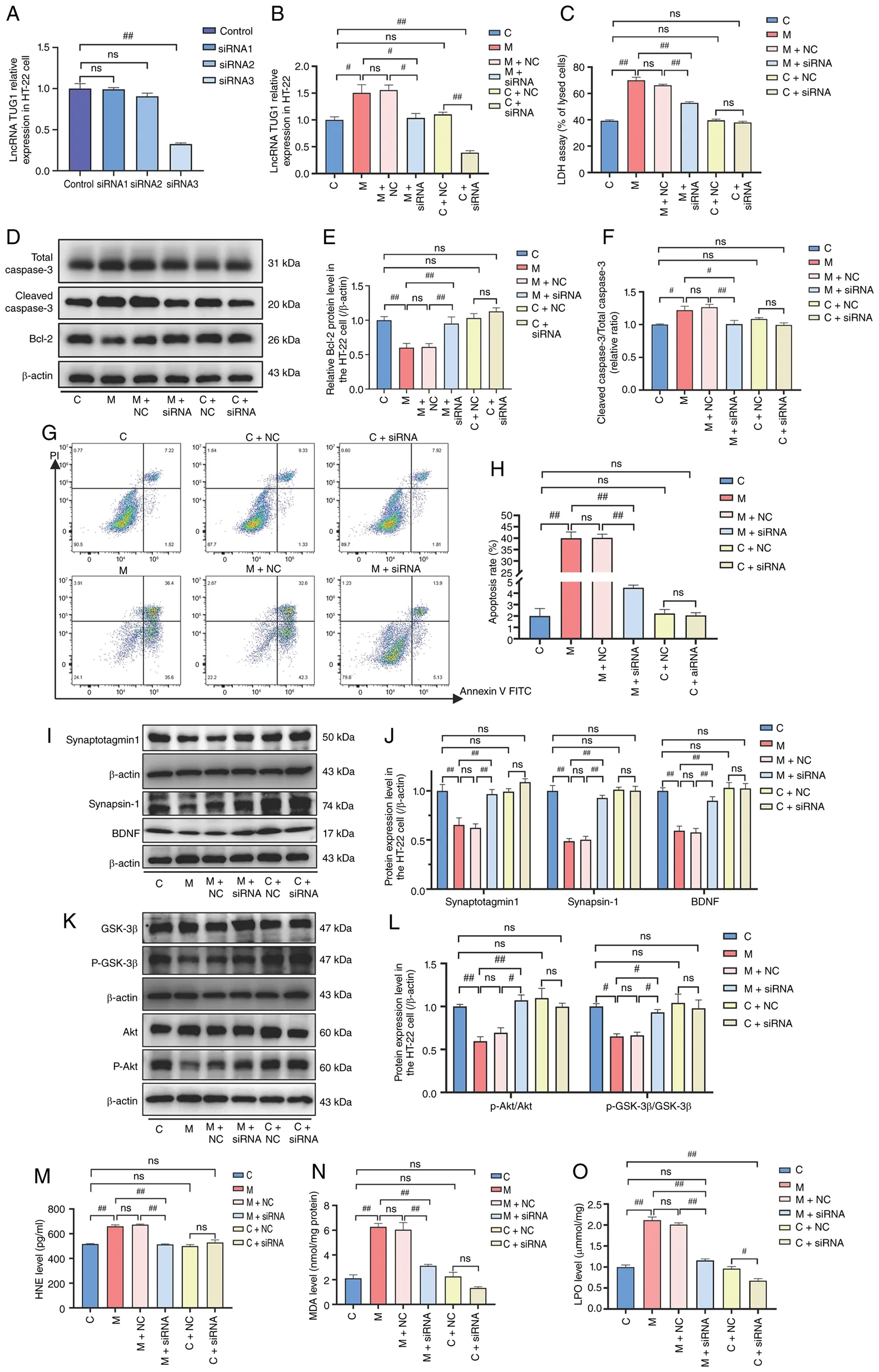
Effects of knocking down lncRNA *TUG1* on apoptosis, synaptic plasticity, the Akt/GSK-3β pathway and LPO levels in PA-challenged HT-22 cells. (A) Determination of lncRNA *TUG1* expression levels in HT-22 cells using different siRNA sequences. (B) Effect of siRNA for lncRNA *TUG1* on the expression level of lncRNA *TUG1* in HT-22 cells with or without PA challenge. (C) LDH release rate assay in si-lncRNA *TUG1* pretreated PA-challenged HT-22 cells. (D) Representative western blotting bands of cleaved caspase-3, total caspase-3 and Bcl-2. Semi-quantification of (E) Bcl-2 protein levels and (F) the cleaved caspase-3 to total caspase-3 ratio. (G) Flow cytometry determination of apoptosis levels in si-lncRNA *TUG1* pretreated PA-challenged HT-22 cells and (H) the statistical analysis results. (I) Representative western blotting bands of Synapsin-1, Synaptotagmin-1 and BDNF. (J) Semi-quantification of Synapsin-1, Synaptotagmin-1 and BDNF protein levels. (K) Representative western blotting bands of Akt, p-Akt, GSK-3β and p-GSK-3β. (L) Semi-quantification of p-Akt/Akt and p-GSK-3β/GSK-3β ratios. Determination of the (M) 4-HNE, (N) MDA levels and (O) LPO levels. The data are presented as the mean ± SEM, with n=3 for each group, except for (H) where n=4 per group. ^#^P<0.05, ^##^P<0.01. 4-HNE, 4-hydroxynonenal; BDNF, brain-derived neurotrophic factor; C, control; LDH, lactate dehydrogenase; lncRNA, long non-coding RNA; LPO, lipid peroxidation; M, model (PA); MDA, malondialdehyde; NC, negative control; ns, not significant; p-, phosphorylated; PA, palmitic acid; si, small interfering (RNA); TUG1, taurine upregulated gene 1.

To knock down lncRNA *TUG1*, three specific siRNAs were designed. Among them, siRNA3 exhibited the most efficient knockdown effect and was selected for subsequent transfection experiments. siRNA3 successfully reduced lncRNA *TUG1* expression in both control and PA-treated cells, whereas the NC showed no significant effect (Fig. 2A and B). The LDH assay showed that lncRNA *TUG1* knockdown significantly reduced PA-induced LDH release compared with the M + NC group, while it did not affect basal LDH release in control cells (C + NC vs. C + siRNA) (Fig. 2C). This indicated that lncRNA *TUG1* knockdown attenuated PA-induced cytotoxicity. lncRNA *TUG1* knockdown also attenuated the PA-induced increase in the cleaved caspase-3 to total caspase-3 ratio and restored Bcl-2 expression (Fig. 2D-F), leading to a reduced apoptotic rate as measured by Annexin V/PI staining (Fig. 2G and H). These results suggested that lncRNA *TUG1* knockdown inhibited apoptosis in PA-treated HT-22 cells. Moreover, the PA-induced downregulation of synaptic proteins including synaptotagmin-1, synapsin-1 and BDNF was alleviated by lncRNA *TUG1* knockdown (Fig. 2I and J), indicating that knocking down lncRNA *TUG1* protected against PA-induced synaptic damage. Consistently, the reduced ratios of p-Akt/Akt and p-GSK-3β/GSK-3β were also restored (Fig. 2K and L), suggesting that lncRNA *TUG1* knockdown reactivated the Akt/GSK-3β signaling pathway. Finally, lncRNA *TUG1* knockdown decreased the PA-induced elevation of the LPO markers 4-HNE, MDA and LPO (Fig. 2M-O), indicating that lncRNA *TUG1* knockdown mitigated LPO.

### miR-449a-5p is a direct target of lncRNA TUG1

**Figure 3:**
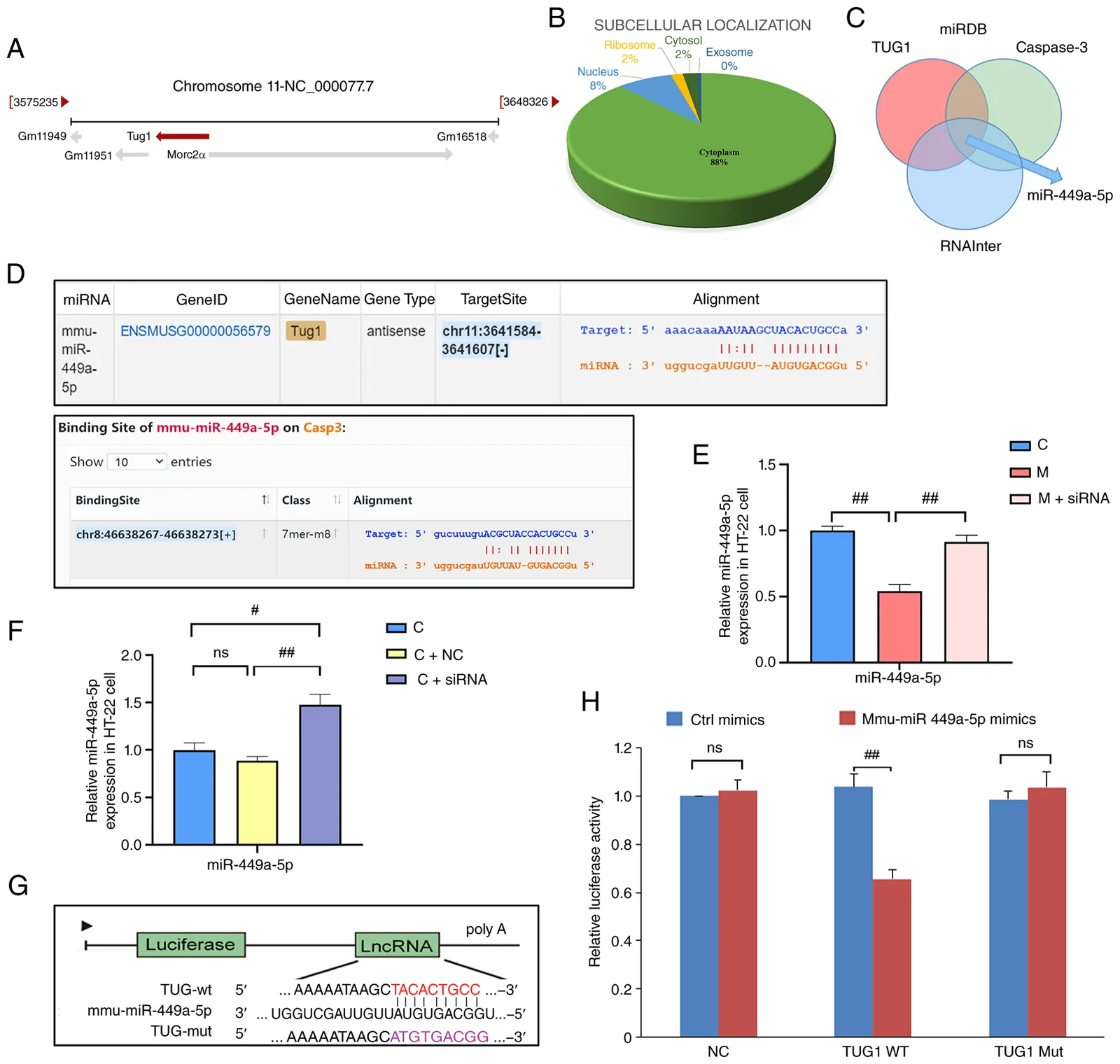
Target relationship between lncRNA *TUG1* and miR-449a-5p. (A) Informational characterization of lncRNA *TUG1* found through the National Center for Biotechnology Information website. (B) Subcellular localization of lncRNA *TUG1* obtained from an online lncLocator predictor. (C) Venn diagram of predicted miRNAs targeting lncRNA and Caspase using miRDB and RNA Interactome databases. (D) Predicted miR-449a-5p binding sites in lncRNA *TUG1* and caspase-3. (E) Relative miR-449a-5p expression level in PA-challenged HT-22 cells pretreated with si-lncRNA *TUG1*. The groups include untreated control cells 'C', the PA-stimulated group 'M', and the PA plus si-lncRNA TUG1 group 'M + siRNA'. (F) Relative miR-449a-5p expression level in HT-22 cells (without PA-challenged) pretreated with si-lncRNA *TUG1*. The groups include untreated control cells 'C', control cells transfected with negative control siRNA 'C + NC' and control cells transfected with si-lncRNA TUG1 'C + siRNA'. (G) Schematic representation of the dual luciferase reporter gene assay of lncRNA *TUG1* with miR-449a-5p. (H) Results of the dual-luciferase reporter assay containing lncRNA *TUG1*-WT/Mut with miR-449a-5p mimic or miR-449a-5p mimic NC. Ctrl mimics are the negative control miRNA mimics (scrambled sequence). The data are presented as the mean ± SEM, with n=3 for each group. ^#^P<0.05, ^##^P<0.01. C, control; Ctrl, control; lncRNA, long non-coding RNA; M, model (PA); miR, microRNA; Mut, mutant; NC, negative control; ns, not significant; PA, palmitic acid; si, small interfering; TUG1, taurine upregulated gene 1; WT, wild-type.

To investigate the function of lncRNA *TUG1*, detailed characteristics from the NCBI database were obtained. As shown in Fig. 3A, this lncRNA is located on chromosome 11. Thereafter, to explore the underlying functional mechanism of lncRNA *TUG1*, the subcellular localization of lncRNA *TUG1* was analyzed using LncLocator 2.0, which showed that it is primarily located in the cytoplasm rather than in the nucleus (Fig. 3B). Given that the ceRNA mechanism is a common regulatory mode for cytoplasmic lncRNAs, this framework was adopted for the further investigation of lncRNA *TUG1*. Caspase-3 is a key executioner of apoptosis and has been reported to be targeted by miRNAs (34). To investigate whether it could participate in the ceRNA regulatory network involving lncRNA *TUG1* under PA-induced HT-22 cell injury, a target prediction search using the miRDB and RNA Interactome databases was performed to identify the target miRNAs for lncRNA *TUG1* and caspase-3. As shown in Fig. 3C, miR-449a-5p had the potential to bind to lncRNA *TUG1* or caspase-3. The potential binding sites between lncRNA *TUG1* and miR-449a-5p, and between caspase-3 and miR-449a-5p were further predicted (Fig. 3D). RT-qPCR was performed to assess the effect of si-lncRNA *TUG1* on the expression of miR-449a-5p in PA-stimulated HT-22 cells. The results showed that the PA-induced reduction in miR-449a-5p expression was alleviated by lncRNA *TUG1* knockdown (P<0.01; Fig. 3E), indicating that lncRNA *TUG1* may negatively regulate miR-449a-5p levels under PA stress. In addition, miR-421 and miR-199a-3p, which have been previously reported to potentially exhibit sponge relationships with lncRNA *TUG1*, as well as miR-6378 and miR-34a-5p, which were predicted to have binding interactions with lncRNA *TUG1*, were detected (Fig. S1M). We hypothesized that lncRNA *TUG1* may repress miR-449a-5p levels through direct binding and molecular sequestration by acting as a sponge that limits miRNA availability or promotes its degradation. To determine whether this regulatory effect is dependent on PA treatment, the effect of *TUG1* knockdown on miR-449a-5p expression under non-PA conditions was examined. Notably, *TUG1* knockdown significantly increased miR-449a-5p expression even in the absence of PA stimulation, thus indicating a basal repressive effect of *TUG1* on this miRNA (Fig. 3F).

To confirm the binding between lncRNA *TUG1* and miR-449a-5p, a dual-luciferase reporter gene assay was performed (Fig. 3G). The results showed that the relative luciferase activity of the lncRNA *TUG1*-WT + miR-449a-5p mimics group was lower than that of the lncRNA *TUG1*-WT + Ctrl mimics group (P<0.01). However, no significant difference was observed when miR-449a-5p mimics or Ctrl mimics were co-transfected with lncRNA *TUG1*-Mut (P>0.05) (Fig. 3H). These findings confirmed that miR-449a-5p may directly bind to the predicted site on lncRNA *TUG1*. On the basis of these results, we hypothesized that miR-449a-5p may serve as a direct target of lncRNA *TUG1* in PA-induced HT-22 cells.

### Overexpression of miR-449a-5p in PA-challenged HT-22 cells alleviates the dysfunctional changes in apoptosis, synaptic plasticity, Akt/GSK-3β pathway and LPO

**Figure 4:**
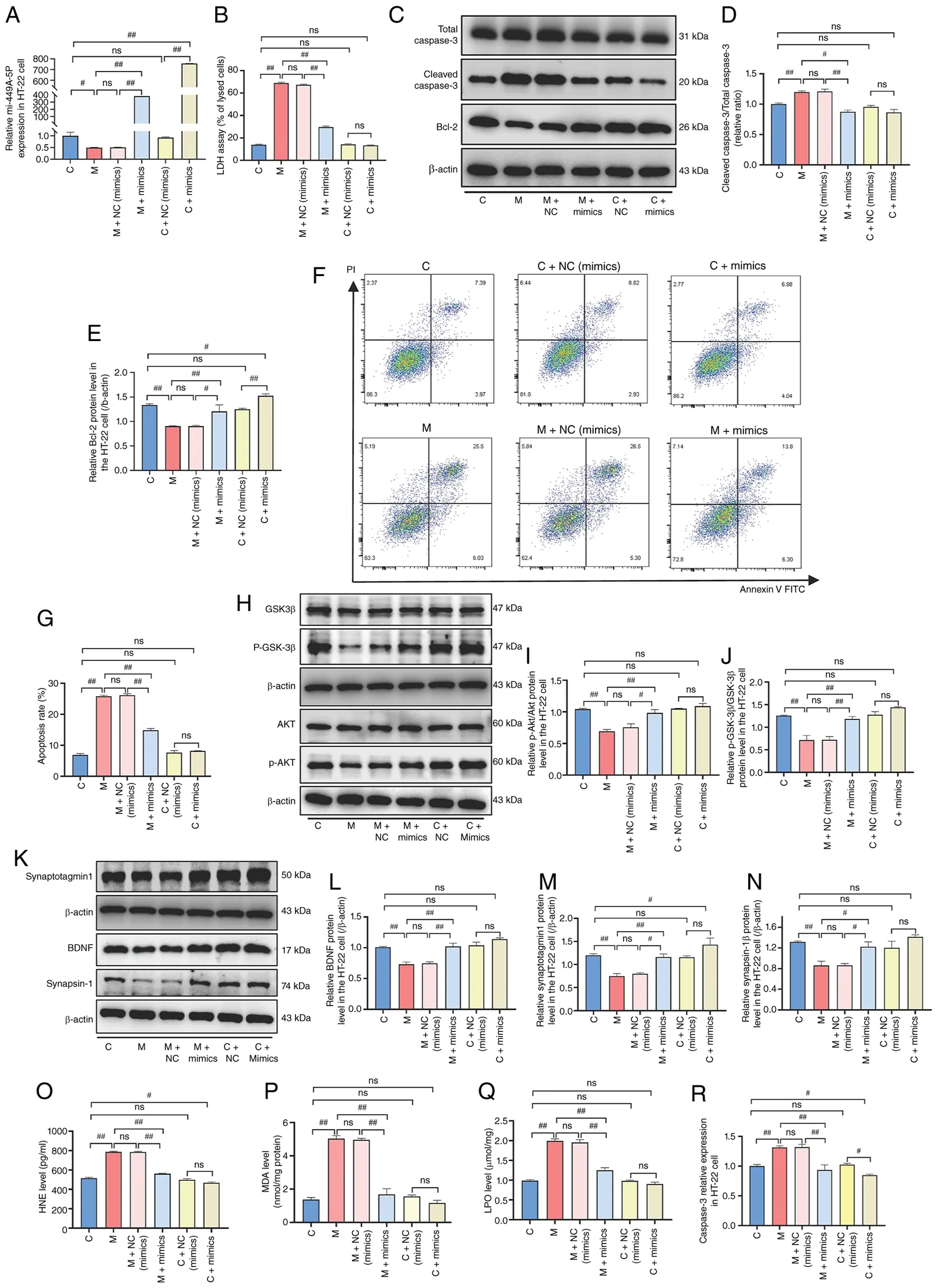
Effects of overexpression of miR-449a-5p on apoptosis, synaptic plasticity, the Akt/GSK-3β pathway and LPO levels in PA-challenged HT-22 cells. (A) Effect of pretreatment with miR-449a-5p mimics on miR-449a-5p expression levels in PA-challenged HT-22 cells. (B) Detection of LDH release rate in PA-challenged HT-22 cells by pretreatment with miR-449a-5p mimics. (C) Representative western blotting bands of Bcl-2, cleaved caspase-3 and total caspase-3. Semi-quantification of (D) the cleaved caspase-3 to total caspase-3 ratio and (E) Bcl-2 protein levels. (F) Flow cytometry determination of apoptosis levels in miR-449a-5p mimic pretreated PA-challenged HT-22 cells. (G) Statistical analysis of apoptosis rates. (H) Representative western blotting bands of Akt, p-Akt, GSK-3β and p-GSK-3β. Semi-quantification of (I) p-Akt/Akt ratio and (J) p-GSK-3β/GSK-3β ratio. (K) Representative western blotting bands of Synapsin-1, Synaptotagmin-1 and BDNF. Semi-quantification of (L) BDNF, (M) Synaptotagmin-1 and (N) Synapsin-1 protein levels. Determination of (O) 4-HNE, (P) MDA and (Q) LPO levels. (R) Effect of pretreatment with miR-449a-5p mimics on caspase-3 expression levels in PA-challenged HT-22 cells. The data are presented as the mean ± SEM, with n=3 for each group, except for (G) where n=4 per group. ^#^P<0.05, ^##^P<0.01. 4-HNE, 4-hydroxynonenal; BDNF, brain-derived neurotrophic factor; C, control; LDH, lactate dehydrogenase; LPO, lipid peroxidation; M, model (PA); MDA, malondialdehyde; miR, microRNA; NC, negative control; ns, not significant; p, phosphorylated; PA, palmitic acid.

To investigate whether miR-449a-5p directly regulates PA-induced neuronal injury, cells were transfected with miR-449a-5p mimics. Efficient transfection was confirmed in Fig. 4A, indicating successful overexpression of miR-449a-5p. The overexpression of miR-449a-5p phenocopied the protective effects of lncRNA *TUG1* knockdown. Specifically, miR-449a-5p mimics significantly reduced PA induced LDH release (Fig. 4B), indicating that miR-449a-5p overexpression attenuated PA-induced cytotoxicity. Furthermore, miR-449a-5p mimics restored Bcl-2 expression and reversed the PA-induced increase in the cleaved caspase-3 to total caspase-3 ratio (Fig. 4C-E). miR-449a-5p mimics also lowered the apoptotic rate, as measured by flow cytometry (Fig. 4F and G), suggesting that miR-449a-5p mimics inhibited apoptosis in PA-challenged HT-22 cells. The PA induced reductions in synapsin-1, synaptotagmin-1 and BDNF were also reversed (Fig. 4K-N), indicating that miR-449a-5p protected against PA-induced synaptic damage, and the p-Akt/Akt and p-GSK-3β/GSK-3β ratios were restored (Fig. 4H-J), suggesting that miR-449a-5p mimics reactivated the Akt/GSK-3β signaling pathway. Moreover, miR-449a-5p overexpression alleviated PA induced LPO, as indicated by the reduced 4-HNE, MDA and LPO levels (Fig. 4O-R), demonstrating that miR-449a-5p mitigated LPO.

### Inhibition of miR-449a-5p in PA-challenged HT-22 cells promotes the dysfunctional changes in apoptosis, synaptic plasticity, Akt/GSK-3β pathway and LPO

**Figure 5:**
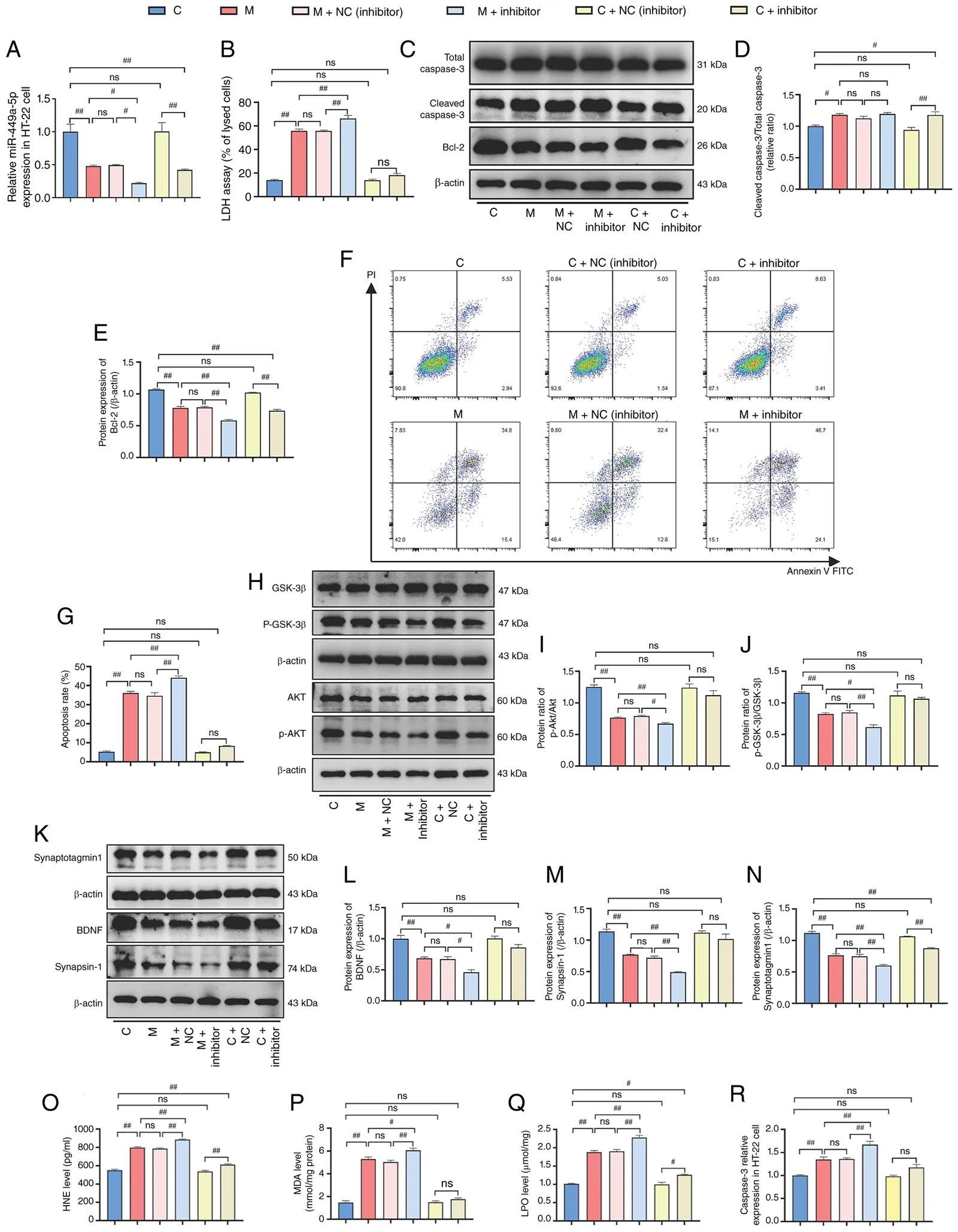
Effects of miR-449a-5p inhibition on apoptosis, synaptic plasticity, Akt/GSK-3β pathway and LPO levels in PA-challenged HT-22 cells. (A) Effect of pretreatment with miR-449a-5p inhibitor on miR-449a-5p expression levels in PA-challenged HT-22 cells. (B) Detection of LDH release rate in PA-challenged HT-22 cells by pretreatment with miR-449a-5p inhibitor. (C) Representative western blotting bands of Bcl-2, cleaved caspase-3 and total caspase-3. Semi-quantification of the (D) cleaved caspase-3 to total caspase-3 ratio and (E) Bcl-2 protein levels. (F) Flow cytometry determination of apoptosis levels in miR-449a-5p inhibitor pretreated PA-challenged HT-22 cells. (G) Statistical analysis of apoptosis rates. (H) Representative western blotting bands of Akt, p-Akt, GSK-3β and p-GSK-3β. Semi-quantification of (I) the p-Akt/Akt and (J) p-GSK-3β/GSK-3β ratios. (K) Representative western blotting bands of Synapsin-1, Synaptotagmin-1 and BDNF. Semi-quantification of the (L) BDNF, (M) Synapsin-1 and (N) Synaptotagmin-1 protein levels. Determination of the (O) 4-HNE, (P) MDA levels and (Q) LPO levels. (R) Effect of pretreatment with miR-449a-5p inhibitor on caspase-3 expression levels in PA-challenged HT-22 cells. The data are presented as the mean ± SEM, with n=3 for each group, except for (G) where n=4 per group. ^#^P<0.05, ^##^P<0.01. 4-HNE, 4-hydroxynonenal; BDNF, brain-derived neurotrophic factor; C, control; LDH, lactate dehydrogenase; LPO, lipid peroxidation; M, model (PA); MDA, malondialdehyde; miR, microRNA; NC, negative control; ns, not significant; p, phosphorylated; PA, palmitic acid.

In contrast to the protective effects observed with miR-449a-5p overexpression, the inhibition of miR-449a-5p using a specific inhibitor exacerbated PA-induced neuronal injury. The inhibitor was efficiently transfected and the negative control showed no significant effect on miR-449a-5p expression (Fig. 5A). LDH release was further increased in PA challenged cells following miR-449a-5p inhibition compared with that in the PA-only or NC groups (Fig. 5B), indicating that miR-449a-5p inhibition aggravated PA-induced cytotoxicity. Transfection with the miR-449a-5p inhibitor increased the cleaved caspase-3 to total caspase-3 ratio in the absence of PA stimulation. In the presence of PA, it did not further alter this ratio but led to a further reduction in Bcl-2 expression (Fig. 5C-E), thus resulting in an increased apoptotic rate, as determined by flow cytometry (Fig. 5F and G). These results suggested that miR-449a-5p inhibition exacerbated apoptosis in PA-challenged HT-22 cells. Moreover, the reductions in the p-Akt/Akt and p-GSK-3β/GSK-3β ratios were further pronounced (Fig. 5H-J), suggesting that miR-449a-5p inhibition further suppressed the Akt/GSK-3β signaling pathway, and the PA-induced downregulation of synaptotagmin-1, synapsin-1 and BDNF was aggravated by miR-449a-5p inhibition (Fig. 5K-N), indicating that loss of miR-449a-5p function worsened PA-induced synaptic damage. Additionally, the inhibition of miR-449a-5p promoted PA-induced elevations in the LPO markers 4-HNE, MDA and LPO (Fig. 5O-Q), indicating that miR-449a-5p deficiency exacerbated LPO.

### lncRNA TUG1/miR-449a-5p axis regulates PA-induced neuronal injury via caspase-3 and the Akt/GSK-3β pathway

**Figure 6:**
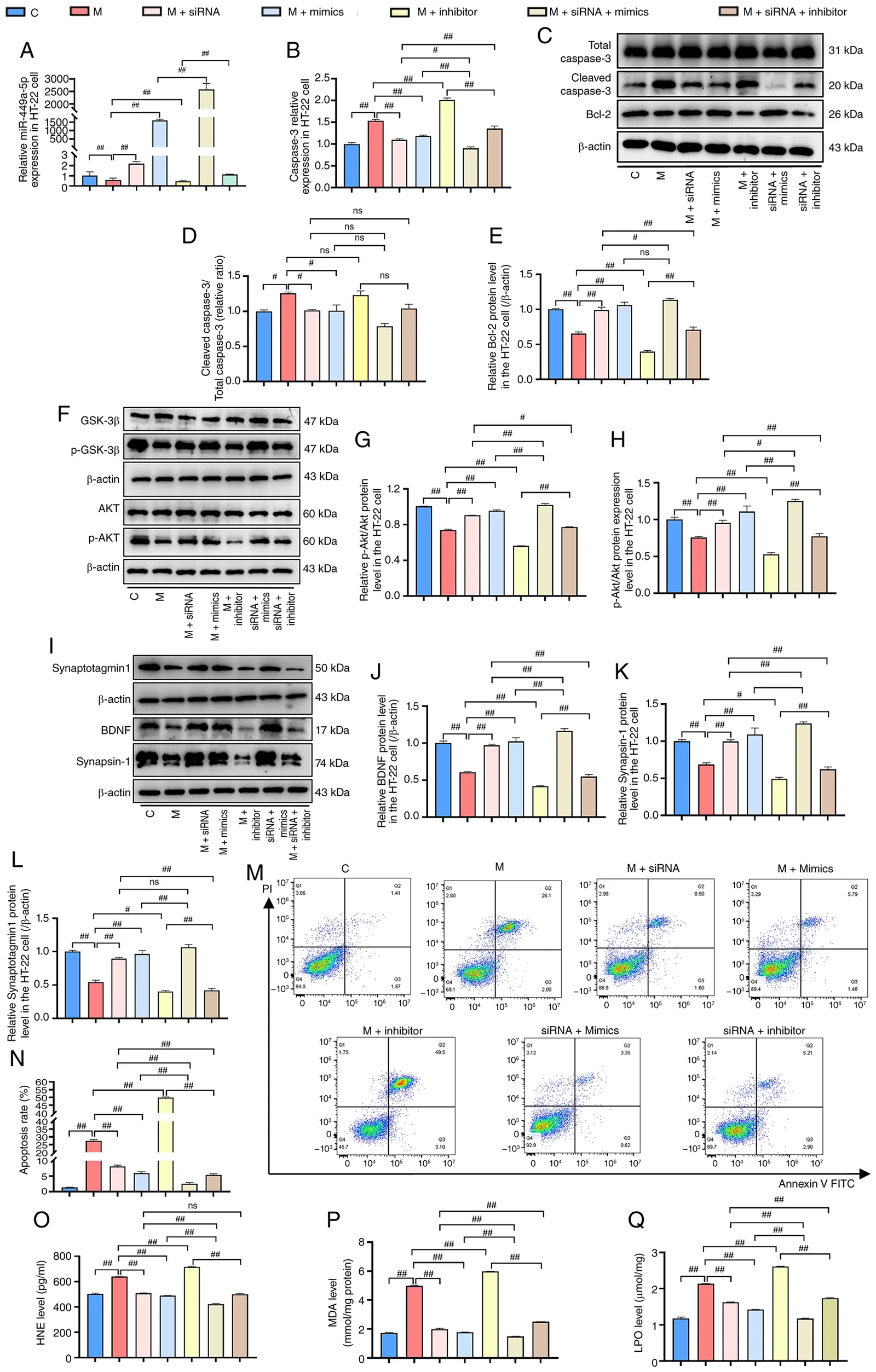
Effect of lncRNA *TUG1*/miR-449a-5p/caspase-3 axis on neuroapoptosis in PA-stimulated HT-22 cells. (A) Relative expression level of miR-449a-5p in PA-stimulated HT-22 cells pretreated with si-lncRNA *TUG1* and/or miR-449a-5p mimics/inhibitor, determined by RT-qPCR. (B) Relative expression level of caspase-3 mRNA. (C) Representative western blot bands of Bcl-2, cleaved caspase-3 and total caspase-3. Semi-quantification of (D) the cleaved caspase-3 to total caspase-3 ratio and (E) Bcl-2 protein levels. (F) Representative western blot bands of Akt, p-Akt, GSK-3β and p-GSK-3β. Semi-quantification of the (G) p-Akt/Akt and (H) p-GSK-3β/GSK-3β ratios. (I) Representative western blot bands of Synapsin-1, Synaptotagmin-1 and BDNF. Semi-quantification of the (J) BDNF, (K) Synaptotagmin-1 and (L) Synapsin-1 protein levels. (M) Flow cytometry determination of the apoptosis levels in PA-stimulated HT-22 cells pretreated as indicated. (N) Statistical analysis of the apoptosis rates. Determination of the (O) 4-HNE, (P) MDA and (Q) LPO levels. The data are presented as the mean ± SEM, with n=3 for each group. ^#^P<0.05, ^##^P<0.01. 4-HNE, 4-hydroxynonenal; BDNF, brain-derived neurotrophic factor; C, control; LDH, lactate dehydrogenase; lncRNA, long non-coding RNA; LPO, lipid peroxidation; M, model (PA); MDA, malondialdehyde; miR, microRNA; ns, not significant; p, phosphorylated; PA, palmitic acid; si, small interfering (RNA); TUG1, taurine upregulated gene 1.

To determine whether lncRNA *TUG1* regulates caspase-3-associated apoptosis by sponging miR-449a-5p, HT-22 cells were co-transfected with si-lncRNA *TUG1* and either miR-449a-5p mimics or an inhibitor prior to PA stimulation. RT-qPCR confirmed that si-lncRNA *TUG1* upregulated miR-449a-5p expression, and this effect was further enhanced by miR-449a-5p mimics. Conversely, the suppressive effect of miR-449a-5p inhibitor was counteracted by si-lncRNA *TUG1* (Fig. 6A). These results confirmed that lncRNA *TUG1* knockdown elevated the miR-449a-5p levels and that the mimic and inhibitor functioned as anticipated. Regarding caspase-3 mRNA levels, compared with the M + siRNA group, caspase-3 expression was increased in the M + siRNA + inhibitor group and decreased in the M + siRNA + mimics group (Fig. 6B), indicating that miR-449a-5p mediates the effect of TUG1 knockdown on caspase-3 transcription. A similar pattern was observed for Bcl-2 protein expression. Compared with the M + siRNA group, Bcl-2 expression was significantly increased in the M + siRNA + mimics group and significantly decreased in the M + siRNA + inhibitor group (Fig. 6C and F). These results suggest that lncRNA *TUG1* regulates Bcl-2 expression, at least partly, through miR-449a-5p. However, under conditions of *TUG1* knockdown, neither the miR-449a-5p inhibitor nor the mimic effectively altered the cleaved caspase-3 to total caspase-3 ratio. The miR-449a-5p inhibitor also reversed the restorative effects of si-lncRNA *TUG1* on p-Akt/Akt and p-GSK-3β/GSK-3β ratios, as well as on synapsin-1, synaptotagmin-1 and BDNF expression. Conversely, the miR-449a-5p mimic enhanced the protective effect of si-lncRNA *TUG1* on Synapsin-1 and BDNF but not on Synaptotagmin-1 (Fig. 6F-L); these findings indicated that the protective effects of si-lncRNA *TUG1* on Akt/GSK-3β signaling and synaptic proteins may be dependent on miR-449a-5p. Flow cytometry further showed that, compared with the M + siRNA group, the apoptosis rate was significantly increased in the M + siRNA + inhibitor group and further decreased in the M + siRNA + mimics group (Fig. 6M and N), further confirming that miR-449a-5p may be essential for the anti-apoptotic effect of lncRNA *TUG1* knockdown. In the LPO assays, the miR-449a-5p inhibitor blocked the ameliorative effect of si-lncRNA *TUG1* on MDA and LPO levels, whereas the mimic potentiated this effect; no significant change was observed for 4-HNE (Fig. 6O-Q), indicating that lncRNA *TUG1* may modulate LPO through miR-449a-5p, with marker-specific variations.

### Pharmacological validation of the Akt/GSK-3β pathway as a downstream effector of the lncRNA TUG1/miR-449a-5p axis

**Figure 7:**
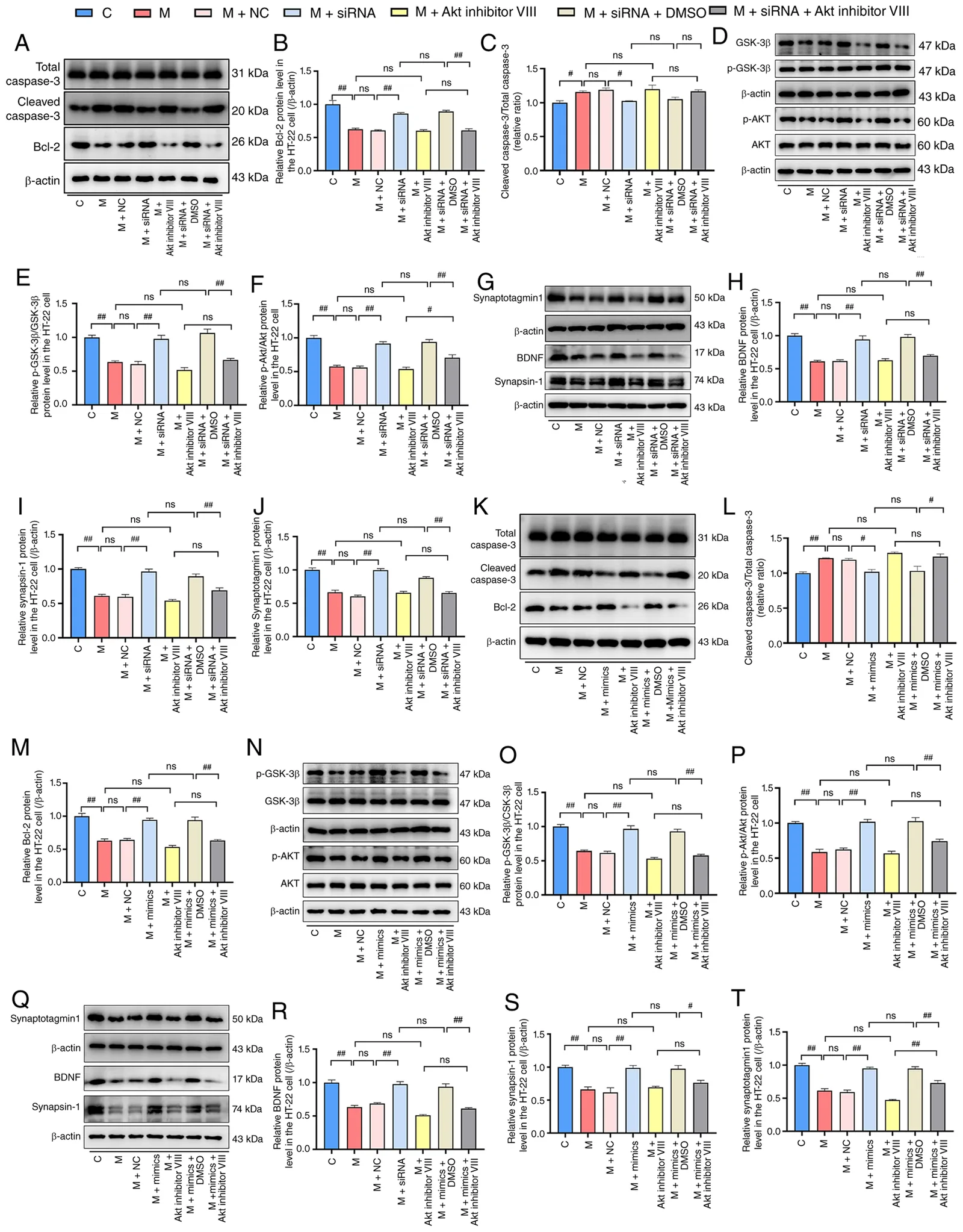
Pharmacological inhibition of Akt abolishes the neuroprotective effects of lncRNA *TUG1* knockdown and miR-449a-5p overexpression in PA-challenged HT-22 cells. (A) Representative western blotting bands of Bcl-2, cleaved caspase-3 and total caspase-3. Semi-quantification of (B) Bcl-2 protein levels and (C) the cleaved caspase-3 to total caspase-3 ratio. (D) Representative western blotting bands of p-Akt, Akt, p-GSK-3β and GSK-3β. Semi-quantification of the (E) p-GSK-3β/GSK-3β and (F) p-Akt/Akt ratios. (G) Representative western blotting bands of Synapsin-1, Synaptotagmin-1 and BDNF. Semi-quantification of the (H) BDNF, (I) Synapsin-1 and (J) Synaptotagmin-1 protein levels. (K) Representative western blotting bands of Bcl-2, cleaved caspase-3 and total caspase-3. Semi-quantification of (L) the cleaved caspase-3 to total caspase-3 ratio and (M) Bcl-2 protein levels. (N) Representative western blotting bands of p-Akt, Akt, p-GSK-3β and GSK-3β. Semi-quantification of the (O) p-GSK-3β/GSK-3β and (P) p-Akt/Akt ratios. (Q) Representative western blotting bands of Synapsin-1, Synaptotagmin-1 and BDNF. Semi-quantification of the (R) BDNF, (S) Synapsin-1 and (T) Synaptotagmin-1 protein levels. The data are presented as the mean ± SEM, with n=3 for each group. ^#^P<0.05, ^##^P<0.01. BDNF, brain-derived neurotrophic factor; C, control; DMSO, dimethyl sulfoxide; lncRNA, long non-coding RNA; M, model (PA); miR, microRNA; NC, negative control; ns, not significant; p, phosphorylated; PA, palmitic acid; siRNA, small interfering RNA; TUG1, taurine upregulated gene 1.

To further verify the critical role of the Akt/GSK-3β pathway in the neuroprotective effects of the lncRNA *TUG1*/miR-449a-5p axis, rescue experiments were performed using a specific pharmacological inhibitor of Akt, namely, Akt inhibitor VIII. Two sets of rescue assays were conducted. In the lncRNA *TUG1* knockdown set, the groups included control (C), model (M; 100 *μ*M PA), M + NC (negative control siRNA), M + siRNA (si-lncRNA *TUG1*), M + Akt inhibitor VIII, M + siRNA + DMSO and M + siRNA + Akt inhibitor VIII. In the miR-449a-5p overexpression set, the groups were C, M, M + NC (mimic NC), M + mimics (miR-449a-5p mimics), M + Akt inhibitor VIII, M + mimics + DMSO and M + mimics + Akt inhibitor VIII. As presented in Fig. 7A-J, compared with the M group, M + Akt inhibitor VIII did not further decrease Bcl-2 protein expression, increase the cleaved caspase-3 to total caspase-3 ratio, significantly affect the p-GSK-3β/GSK-3β and p-Akt/Akt ratios or further reduce the protein expression of Synapsin-1, Synaptotagmin-1 and BDNF. However, compared with the M + siRNA + DMSO group, the M + siRNA + Akt inhibitor VIII group reversed the protective effects of lncRNA *TUG1* knockdown, as evidenced by decreased Bcl-2 protein expression, reduced p-GSK-3β/GSK-3β and p-Akt/Akt ratios and decreased protein expression of Synapsin-1, Synaptotagmin-1 and BDNF. Notably, no significant difference was observed in the cleaved caspase-3 to total caspase-3 ratio. These results further confirmed that the neuroprotective effects of lncRNA *TUG1* knockdown may depend on the functional activation of the Akt/GSK-3β signaling pathway. Similarly, inhibition of Akt reversed the protective effects of miR-449a-5p overexpression, indicating that the protective actions of miR-449a-5p also require the Akt/GSK-3β pathway. Specifically, compared with the M + mimics + DMSO group, the M + mimics + Akt inhibitor VIII group exhibited an increased cleaved caspase-3 to total caspase-3 ratio, decreased Bcl-2 protein expression, reduced p-GSK-3β/GSK-3β and p-Akt/Akt ratios, and decreased protein expression of Synapsin-1, Synaptotagmin-1 and BDNF (Fig. 7K-T). No statistically significant differences were detected in any of the measured parameters between the M + siRNA group and its corresponding DMSO vehicle control group (M + siRNA + DMSO) or between the M + mimics group and its DMSO control (M + mimics + DMSO) (Fig. 7A-T), demonstrating that the vehicle DMSO did not interfere with the observed biological effects. Collectively, these data provide direct pharmacological evidence that the Akt/GSK-3β signaling pathway may be indispensable for the neuroprotective functions of the *TUG1*/miR-449a-5p axis in PA-induced neuronal injury.

## Discussion

The present study identified lncRNA *TUG1* as a potential critical mediator of PA-induced neuronal lipotoxicity via the miR-449a-5p/caspase-3 axis together with the Akt/GSK-3β pathway. Elevated lncRNA *TUG1* expression was detected in PA-treated neuronal cells and was accompanied by increased cytotoxicity, apoptosis and synaptic protein loss. lncRNA *TUG1* knockdown alleviated PA-induced cytotoxicity, apoptosis and synaptic protein loss, while restoring Akt/GSK-3β phosphorylation. Mechanistically, lncRNA *TUG1* acted as a sponge for miR-449a-5p, thus leading to the derepression of caspase-3 expression. The pharmacological inhibition of Akt confirmed that the Akt/GSK-3β pathway may be a required downstream partner of this axis. These findings suggest a detrimental role for lncRNA *TUG1* in lipid-induced neurotoxicity through a regulatory network involving both caspase-3 and Akt/GSK-3β signaling.

A growing body of evidence implicates dysregulated lipid metabolism in the pathogenesis of cognitive decline and neurodegenerative disorders (1,35). Hyperlipidemia, a hallmark of metabolic syndrome and T2DM, is independently associated with an increased risk of all-cause dementia and AD (1,2). Mechanistically, saturated free fatty acids such as PA, which are elevated in the cerebrospinal fluid of patients with obesity and cognitive impairment, can directly trigger neuronal lipotoxicity via multiple pathways, including oxidative stress, endoplasmic reticulum stress, mitochondrial dysfunction and autolysosomal impairment (36). In the central nervous system, PA disrupts autolysosomal function and mitochondrial homeostasis, thus triggering oxidative stress and neuroinflammatory responses, which ultimately contribute to neurodegeneration (37). Furthermore, PA induces dynamic alterations in histone deacetylases and chromatin acetylation in neurons, thus suggesting that lipid overload may have lasting effects on neuronal gene expression beyond acute metabolic stress (2). Consistent with these findings, our previous studies using NAFLD and T2DM animal models have demonstrated that systemic dyslipidemia is accompanied by AD-like neuropathological changes in the hippocampus, including impaired synaptic plasticity, microglial activation and tau hyperphosphorylation (9,38). The present study further demonstrated that PA stimulation alone is sufficient to recapitulate the core features of metabolic neuropathy in an *in vitro* model, including the downregulation of synaptic proteins, exacerbation of apoptosis and dysregulation of the Akt/GSK-3β signaling pathway. Collectively, these observations underscore the notion that lipid-induced neurotoxicity represents a critical yet incompletely understood pathogenic link between peripheral metabolic dysfunction and central nervous system impairment; moreover, PA is not merely a metabolic byproduct but rather an active signaling molecule that mediates the detrimental effects of systemic hyperlipidemia on brain function.

lncRNA *TUG1* has emerged as a pivotal regulatory molecule in the nervous system, participating in a wide array of physiological and pathological processes ranging from neuronal differentiation to neurodegeneration. Accumulating evidence has documented the aberrant expression of lncRNA *TUG1* in multiple neurological disorders. In ischemic stroke models, lncRNA *TUG1* expression is markedly elevated in the ischemic penumbra, and its knockout or knockdown confers neuroprotection by attenuating neuronal apoptosis and suppressing microglial pyroptosis (39,40). Similarly, elevated lncRNA *TUG1* levels have been detected in the serum or cerebrospinal fluid of patients with temporal lobe epilepsy, Parkinson's disease and AD, and its expression has been correlated with disease severity and poor prognosis (18,19). In the context of metabolic dysregulation, lncRNA *TUG1* has been implicated in diabetic cardiomyopathy and NAFLD-associated hepatic injury (17,21). However, its expression profile and functional significance in lipid-induced neuronal injury remain unclear. In the present study, it was found that lncRNA *TUG1* was robustly upregulated in both HT-22 and SH-SY5Y neuronal cells following PA challenge, and that the magnitude of this upregulation was significantly correlated with the severity of lipotoxic injury. More specifically, Pearson correlation analysis revealed that lncRNA *TUG1* expression was positively associated with cleaved caspase-3 levels and LDH release while exhibiting a negative association with anti-apoptotic protein Bcl-2, synaptic plasticity markers including synapsin-1, synaptotagmin-1 and BDNF as well as the Akt and GSK-3β phosphorylation ratios. Although the limited sample size precludes definitive causal conclusions, these exploratory analyses provide a statistical framework for prioritizing downstream effectors and guiding subsequent functional validation and offer preliminary clues suggesting that lncRNA *TUG1* may functionally participate in the regulation of neuronal apoptotic signaling networks under lipotoxic conditions. Taken together, these findings suggest that lncRNA *TUG1* is a potential molecular sensor or amplifier of PA-induced neuronal injury and justify its selection as the central focus of mechanistic investigations.

Neuronal apoptosis and synaptic loss are two inextricably linked pathological hallmarks of metabolic cognitive impairment. Postmortem analyses of hippocampal tissue from patients with amnestic mild cognitive impairment have revealed significantly decreased levels of PSD95, a key postsynaptic scaffolding protein, alongside elevated caspase-3 expression, thus suggesting that coordinated dysregulation of synaptic integrity and apoptotic signaling occurs early in the trajectory of cognitive decline (41). In the context of lipotoxicity, PA induces localized caspase-3 activation that extends beyond its canonical role in cell death. Emerging evidence indicates that caspase-3 can be activated at sublethal levels within the dendritic spines, where it participates in the cleavage of critical synaptic scaffolding proteins and contributes to the weakening of synaptic transmission (42,43). This non-apoptotic function of caspase-3 provides a mechanistic framework for understanding how metabolic stressors such as PA can impair synaptic plasticity and cognitive performance before overt neuronal loss becomes detectable, a phenomenon that aligns with early memory deficits frequently observed in patients with T2DM and NAFLD (37,44). The findings of the present study extend this paradigm by demonstrating that lncRNA *TUG1* functions as an upstream regulator that sustains elevated caspase-3 expression under lipotoxic conditions. By sponging miR-449a-5p and relieving its repression of caspase-3, lncRNA *TUG1* amplifies both the apoptotic cascade and synaptic dysfunction triggered by PA. This dual effect on neuronal survival and synaptic integrity explains why lncRNA *TUG1* expression correlated strongly with both cleaved caspase-3 levels and the depletion of synaptic proteins such as synapsin-1, synaptotagmin-1 and BDNF in the PA model. Furthermore, it provides a molecular rationale for the well-documented association between hyperlipidemia and accelerated cognitive impairment. This caspase-3-mediated dual damage is further exacerbated by the concurrent dysregulation of the Akt/GSK-3β survival pathway, which is also under the control of the lncRNA *TUG1*/miR-449a-5p axis.

In addition, the present study demonstrated that lncRNA *TUG1* also acts as a critical mediator in PA-induced neuronal injury through the Akt/GSK-3β signaling pathway. This pathway may serve as a molecular link between peripheral metabolic dysregulation and central nervous system impairment. To explore the functional role of the Akt/GSK-3β pathway, pharmacological experiments using Akt inhibitor VIII were performed. The results showed that Akt activation may be essential for the neuroprotective effects conferred by both lncRNA *TUG1* knockdown and miR-449a-5p upregulation. In mediating PA-induced lipotoxic injury, the Akt/GSK-3β pathway functions as an important synergistic partner with caspase-3. The crosstalk between caspase-3 and the Akt/GSK-3β pathway has been documented in previous studies. Akt can phosphorylate and inactivate caspase-9, thereby suppressing caspase-3 activation and promoting cell survival (45). Conversely, active caspase-3 can cleave and inactivate Akt, thereby forming a feedback loop that amplifies apoptotic signals (46). GSK-3β is a well-recognized downstream target of Akt; Akt mediated phosphorylation at Ser9 inactivates GSK-3β, and the dysregulation of GSK-3β has been implicated in various neurodegenerative conditions (47). In the context of lipotoxic injury, a previous study has shown that saturated fatty acids can simultaneously trigger caspase-3 activation and suppress Akt signaling, ultimately leading to neuronal apoptosis (48). On the basis of the experimental data from the present study together with these previous reports, we propose that within the lncRNA *TUG1*/miR-449a-5p regulatory axis, caspase-3 and the Akt/GSK-3β pathway do not operate in a simple linear cascade but rather constitute an integrated signaling network in which they mutually influence each other to orchestrate neuronal injury.

The findings of the present study hold potential clinical relevance in metabolic neuropathy and T2DM related neurodegeneration. T2DM is a well-recognized risk factor for cognitive impairment and dementia. Recent meta-analyses have demonstrated that individuals with diabetes have a significantly higher risk of developing dementia compared with those without diabetes, with factors such as glycemic control and disease duration showing significant associations with cognitive outcomes (49,50). Among the various pathogenic factors linking T2DM to brain dysfunction, lipotoxicity resulting from elevated levels of saturated fatty acids, such as PA, is considered a key contributor to neuronal injury and synaptic loss. Emerging evidence indicates that PA disrupts autolysosomal function and mitochondrial homeostasis, triggering oxidative stress and neuroinflammatory responses that ultimately lead to neurodegeneration (37). The present study revealed that lncRNA *TUG1* may act as a critical mediator in PA-induced neuronal lipotoxicity through the miR-449a-5p/caspase-3 axis together with the Akt/GSK-3β signaling pathway. This pathway may serve as a molecular link between peripheral metabolic dysregulation and central nervous system impairment. Notably, elevated levels of lncRNA *TUG1* have been detected in both the peripheral circulation and cerebrospinal fluid of patients with neurological disorders, and these levels are negatively correlated with patient prognosis (51-53). This observation suggests that lncRNA *TUG1* holds promise as a potential biomarker for early diagnosis or disease progression assessment in T2DM associated cognitive decline. Furthermore, the neuroprotective effects observed following lncRNA *TUG1* knockdown or miR-449a-5p overexpression *in vitro* in the present study indicated that targeting this regulatory axis may offer a novel therapeutic strategy for neuropsychiatric complications in patients with metabolic disorders. Although further validation in animal models and clinical samples is warranted, the present study provides a molecular framework for developing intervention strategies that are aimed at preventing or ameliorating T2DM related neurodegeneration.

Although the present study elucidated the novel role and underlying mechanisms of the lncRNA *TUG1*/miR-449a-5p/caspase-3 axis in PA-induced neuronal apoptosis, several limitations should be acknowledged. First, the experimental findings were primarily derived from immortalized neuronal cell lines, namely, HT-22 and SH-SY5Y cells. Although these cell lines are widely accepted and frequently used in neurobiological research due to their accessibility and experimental reproducibility, they do not fully recapitulate the complex physiological properties of primary neurons. Primary neurons maintain intact synaptic connectivity, exhibit more physiologically relevant electrophysiological characteristics and may respond to metabolic stress in ways that differ substantially from those of immortalized cell lines. Therefore, the absence of validation in primary cultured neurons is a limitation of the present study. Second, some of the experiments were conducted with relatively small sample sizes. Although the results were highly consistent across multiple independent assays, reducing the risk of false-positive results, larger sample sizes would further improve the robustness and reproducibility of the conclusions drawn. Third, although bioinformatics predictions and dual-luciferase reporter assays supported the direct targeting of miR-449a-5p and both lncRNA *TUG1* and caspase-3, additional biochemical experiments such as RNA pull-down or argonaute 2-based RNA immunoprecipitation assays were not performed in the present study. Such experiments would provide further support for the direct interactions. Therefore, the current evidence, which is consistent with our proposed model, does not entirely exclude the possibility of indirect regulation. Future studies employing these approaches will help solidify this conclusion. Fourth, no loss of function or gain of function interventions were performed *in vivo*, such as stereotaxic injection of lncRNA *TUG1* specific siRNA or overexpression vectors into the hippocampus, followed by an assessment of cognitive or behavioral changes in animals. Consequently, the causal role of the lncRNA *TUG1*/miR-449a-5p/caspase-3 signaling axis in T2DM related neurodegeneration *in vivo* has not yet been established. Furthermore, it was not validated whether targeting this pathway could ameliorate cognitive deficits or neuropathological changes in the animal models. These missing *in vivo* experiments limit the translational relevance of the present work. Fifth, although it was confirmed that the lncRNA *TUG1*/miR-449a-5p axis may regulate caspase-3-dependent neuronal apoptosis by detecting cleaved caspase-3 expression, Bcl-2 levels, LDH release and flow cytometry, TUNEL or Hoechst staining to provide direct morphological evidence of apoptosis was not performed, nor was poly(ADP-ribose) polymerase cleavage detected or mitochondrial membrane potential measured in the present study. These indicators provide important supplementary evidence for the mitochondrial apoptotic pathway and further confirm the role of caspase-3 as a key downstream effector. This is another limitation of the present study.

In conclusion, the present study demonstrated that lncRNA *TUG1* was upregulated in PA-induced neuronal injury and may participate in lipotoxic neuronal damage through the miR-449a-5p/caspase-3 axis together with the Akt/GSK-3β signaling pathway. The findings also revealed a role for lncRNA *TUG1* in PA-induced neuronal lipotoxicity, a pathological process closely associated with hyperlipidemia-related neuronal damage. In addition, miR-449a-5p was identified as a novel target of lncRNA *TUG1* in the regulation of lipotoxicity-associated neuronal apoptosis and the findings confirmed that miR-449a-5p can modulate the expression of caspase-3. These results provide experimental evidence for understanding the pathogenesis of neuropsychiatric dysfunction linked to lipid metabolism disorders and offer preliminary clues for exploring lncRNA *TUG1* as a potential therapeutic target.

## Supplementary Data



## Data Availability

The data generated in the present study may be requested from the corresponding author.

## Abbreviations

4-HNE: 4-hydroxynonenal
AD: Alzheimer's disease
BDNF: brain-derived neurotrophic factor
ceRNA: competing endogenous RNA
LDH: lactate dehydrogenase
lncRNAs: long non-coding RNAs
miR: microRNA
NAFLD: non-alcoholic fatty liver disease
NCBI: National Center for Biotechnology Information
PA: palmitic acid
RT-qPCR: reverse transcription-quantitative polymerase chain reaction
siRNA: small interfering RNA
T2DM: type 2 diabetes mellitus
TUG1: taurine upregulated gene 1
