# Supplementary material for: lncRNA TUG1 mediates palmitic acid-induced neuronal lipotoxic injury via the miR-449a-5p/caspase-3 and Akt/GSK-3β axes
Source: Int J Mol Med. 2026 Aug 25;58(5):295. doi: 10.3892/ijmm.2026.5966 (PMC13541273; doi:10.3892/ijmm.2026.5966)

Figure S1. Effects of PA challenge on lncRNA *TUG1* expression as well as apoptosis, synaptic plasticity and Akt/GSK-3β pathway in SH-SY5Y cells. (A) MTT results of SH-SY5Y cells challenged with different concentrations of PA. (B) Expression levels of lncRNA *TUG1* in SH-SY5Y cells. (C) Representative western blotting bands of Akt, p-Akt, GSK-3β and p-GSK-3β. Semi-quantification of the (D) p-Akt/Akt and (E) p-GSK-3β/GSK-3β ratios. (F) Representative western blotting bands of Bcl-2, cleaved caspase-3 and total caspase-3. Semi-quantification of the (G) cleaved caspase-3 to total caspase-3 ratio and (H) Bcl-2 protein levels. (I) Representative western blotting bands of Synapsin-1, Synaptotagmin-1 and BDNF. Semi-quantification of the (J) BDNF, (K) Synapsin-1 and (L) Synaptotagmin-1 protein levels. (M) miRNA expression levels in HT-22 cells. The data are presented as the mean ± SEM, with n=3 for each group. #P<0.05, ##P<0.01 compared with the control group. BDNF, brain-derived neurotrophic factor; C, control; lncRNA, long non-coding RNA; M, model (PA); miR, microRNA; MTT, 3-(4,5-dimethylthiazol-2-yl)-2,5-diphenyltetrazolium bromide assay; ns, not significant; p-, phosphorylated; PA, palmitic acid; TUG1, taurine upregulated gene 1.

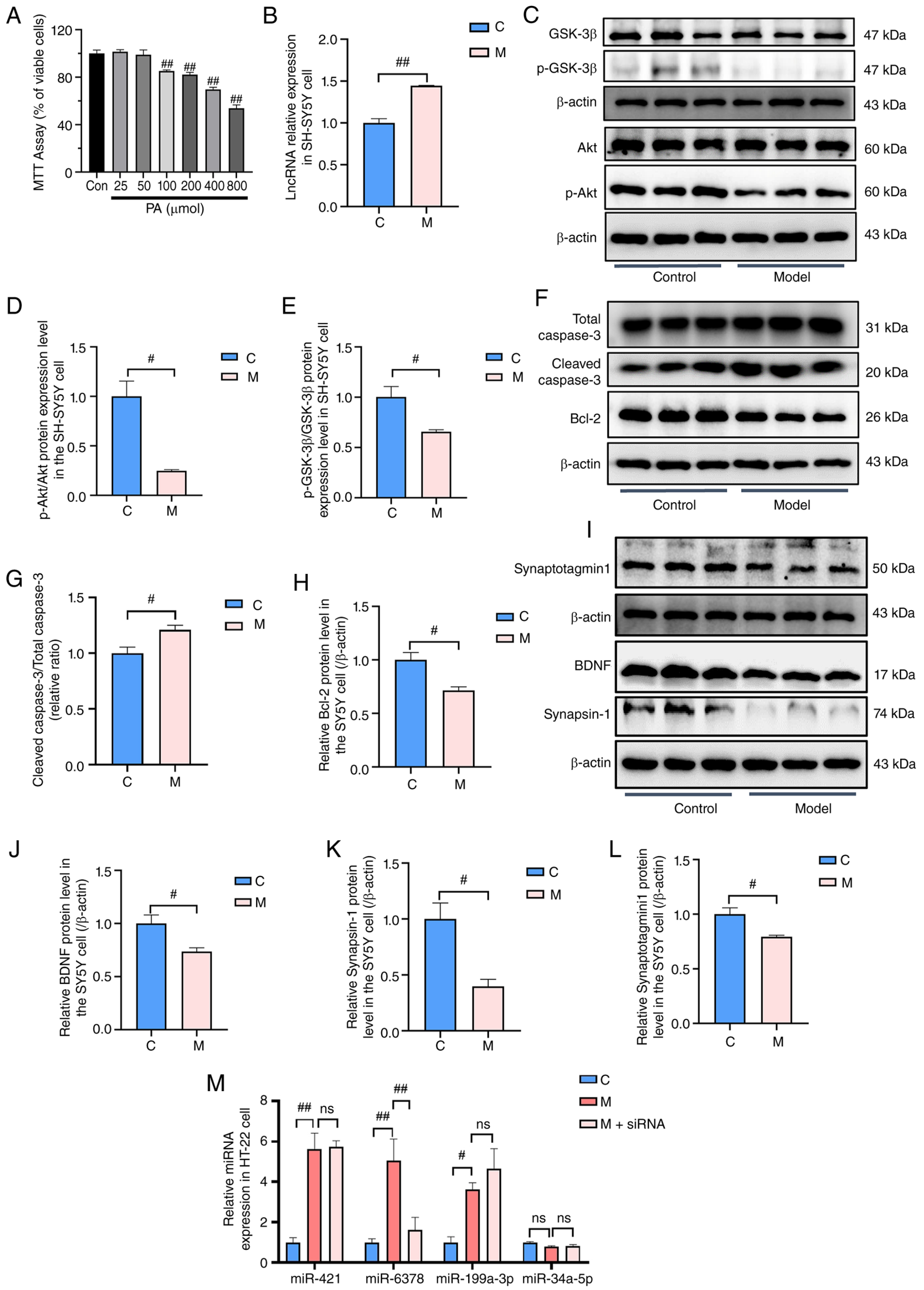

Supplement: Supplementary file 1 [file IJMM-58-5-05966-Supplementary_Data1.pdf]
