# Supplementary material for: lncRNA TUG1 mediates palmitic acid-induced neuronal lipotoxic injury via the miR-449a-5p/caspase-3 and Akt/GSK-3β axes
Source: Int J Mol Med. 2026 Aug 25;58(5):295. doi: 10.3892/ijmm.2026.5966 (PMC13541273; doi:10.3892/ijmm.2026.5966)
Supplement: Supplementary file 2 [file IJMM-58-5-05966-Supplementary_Data2.pdf]

>*TUG1* WT

Agactgctcacagttaacaaatcaaactctggatgacttctgcttgtatatgcaactact
atttgtaaagaaattgcaaattcacttttctattacctctacattgctagctctttcttttgt
gtttgtattaaaaacaaaaataagc**tacactgcc**agctattccctcctgccatactcagt
taaaatgaagaatcgggaatctaaccagtgaatggataagtagaaaaaactaaaactt
aaggcaaaagccttaatctagggccttttctactatcttcatgtcttggatttcatcta

>*TUG1* MUT

agactgctcacagttaacaaatcaaactctggatgacttctgcttgtatatgcaactact
atttgtaaagaaattgcaaattcacttttctattacctctacattgctagctctttcttttgt
gtttgtattaaaaacaaaaataagc**atgtgacgg**agctattccctcctgccatactc
agttaaaatgaagaatcgggaatctaaccagtgaatggataagtagaaaaaactaaa
acttaaggcaaaagccttaatctagggccttttctactatcttcatgtcttggatttcatcta

Sequence annotation: The underlined and bold segment in the *TUG1* WT sequence represents the original nucleotide sequence. The corresponding underlined and bold region in the *TUG1* MUT sequence contains site-directed nucleotide substitutions to construct the mutated fragment. MUT, mutant; TUG1, taurine upregulated gene 1; WT, wild-type.
